# The plasma proteome is favorably modified by a high protein diet but not by additional resistance training in older adults: A 17-week randomized controlled trial

**DOI:** 10.3389/fnut.2022.925450

**Published:** 2022-08-05

**Authors:** Bernhard Franzke, Andrea Bileck, Sandra Unterberger, Rudolf Aschauer, Patrick A. Zöhrer, Agnes Draxler, Eva-Maria Strasser, Barbara Wessner, Christopher Gerner, Karl-Heinz Wagner

**Affiliations:** ^1^Research Platform Active Ageing, University of Vienna, Vienna, Austria; ^2^Department of Nutritional Sciences, Faculty of Life Sciences, University of Vienna, Vienna, Austria; ^3^Department of Analytical Chemistry, Faculty of Chemistry, University of Vienna, Vienna, Austria; ^4^Joint Metabolome Facility, University of Vienna and Medical University of Vienna, Vienna, Austria; ^5^Centre for Sport Science and University Sports, University of Vienna, Vienna, Austria; ^6^Karl Landsteiner Institute for Remobilization and Functional Health/Institute for Physical Medicine and Rehabilitation, Kaiser Franz Joseph Hospital, Social Medical Center South, Vienna, Austria

**Keywords:** plasma proteomics, healthy aging, strength training, high protein foods, innate immune system, lipid transport, blood coagulation system, life style intervention

## Abstract

**Background:**

The age-related loss of muscle mass significantly contributes to the development of chronic diseases, loss of mobility and dependency on others, yet could be improved by an optimized lifestyle.

**Objective:**

The goal of this randomized controlled trial was to compare the influence of a habitual diet (CON) with either a diet containing the recommended protein intake (RP) or a high protein intake (HP), both with and without strength training, on the plasma proteome in older adults.

**Methods:**

One hundred and thirty-six women and men (65–85 years) were randomly assigned to three intervention groups. CON continued their habitual diet; participants of the HP and RP group consumed either high protein or standard foods. After 6 weeks of dietary intervention, HP and RP groups additionally started a strength training intervention twice per week for 8 weeks. Twenty-four hours dietary recalls were performed every 7–10 days. Body composition was assessed and blood taken. Plasma proteomics were assessed with LC-MS.

**Results:**

Participants of the HP group doubled their baseline protein intake from 0.80 ± 0.31 to 1.63 ± 0.36 g/kg BW/d; RP increased protein intake from 0.89 ± 0.28 to 1.06 ± 0.26 g/kg BW/d. The CON group kept the protein intake stable throughout the study. Combined exercise and HP initiated notable changes, resulting in a reduction in bodyfat and increased muscle mass. Proteomics analyses revealed 14 significantly affected proteins by HP diet, regulating innate immune system, lipid transport and blood coagulation, yet the additional strength training did not elicit further changes.

**Conclusions:**

Combined HP and resistance exercise in healthy older adults seem to induce favorable changes in the body composition. Changes in the plasma proteome due to the high protein diet point to a beneficial impact for the innate immune system, lipid transport and blood coagulation system, all of which are involved in chronic disease development.

**Clinical trial registration:**

The study was registered at ClinicalTrials.gov (NCT04023513).

## Introduction

We are living in a rapidly aging society. Prognosis from the WHO expect the proportion of people with an age over 60 years to double between 2015 and 2050. Specifically the group of the very old (80 years or older) will triple within the same timeframe (1). The resulting shift toward an even higher population age will increase the already high healthcare costs and further raise the socio-economic burden (2).

Aging is associated with an increased risk for most chronic diseases—such as type 2 diabetes, cancer, cardiovascular diseases or dementia—as well as dependence on other people, all of which could be linked to a loss of and/or reduced physical and physiological capacity (3). A higher proportion of muscle mass in advanced age is linked to better quality of life, reduced risk for metabolic disfunction/disease and improved outcomes after diseases and therapeutic interventions (4, 5). To counteract the aging-related loss of muscle mass a healthy lifestyle, including regular physical exercise and an appropriate diet is beneficial and cost-effective on an individual level as well as on population scale. The most effective non-pharmaceutical way to increase and or maintain muscle mass in older adults is the combination of physical activity (specifically strength-oriented exercises) and an optimized protein intake (6, 7). Current recommendations regarding protein intake are critically discussed and challenged to be increased, specifically in the context of muscle hypertrophy and aging (8). Noteworthy, the aging muscle's response mechanisms after an exercise and/or nutritional stimulus are significantly reduced compared to younger adults (9, 10). More and more data pointing toward the direction, that this so-called anabolic resistance could, at least to some extent, be compensated by increasing the protein intake to about twice the amount as necessary for younger adults, to equally stimulate muscle protein synthesis in older adults (11, 12).

To explore the complex metabolic interplay between nutrition and exercise training, the so-called omics-methods are being used, such as genomics, transcriptomics, proteomics or metabolomics (13–15). Within these methods, proteomics aims at identifying specific proteins or protein expression patterns to possibly link clinical outcomes to proteomic profiles (16). Yet, data regarding resistance exercise or high protein diet interventions in older adults in the context of proteomics profiling is very limited (17, 18) and the combination is even more rare.

We hypothesized that doubling the protein intake of older adults would induce greater changes in the plasma proteome and would be superior to induce changes in body composition, as compared to a habitual diet and/or a protein intake according to the recommendations for this age group. A combined intervention of diet and strength training would lead to even stronger adaptations, which would also be reflected by more distinct changes in the plasma proteome.

Therefore, the goal of this randomized controlled trial was to compare the influence of a habitual diet with either a diet containing the recommended protein intake or a high protein intake, both with and without strength training, on the plasma proteome (as secondary outcome analysis) and link it to body composition parameters in community-dwelling older adults.

## Materials and methods

### Study design

**Figure 1:**
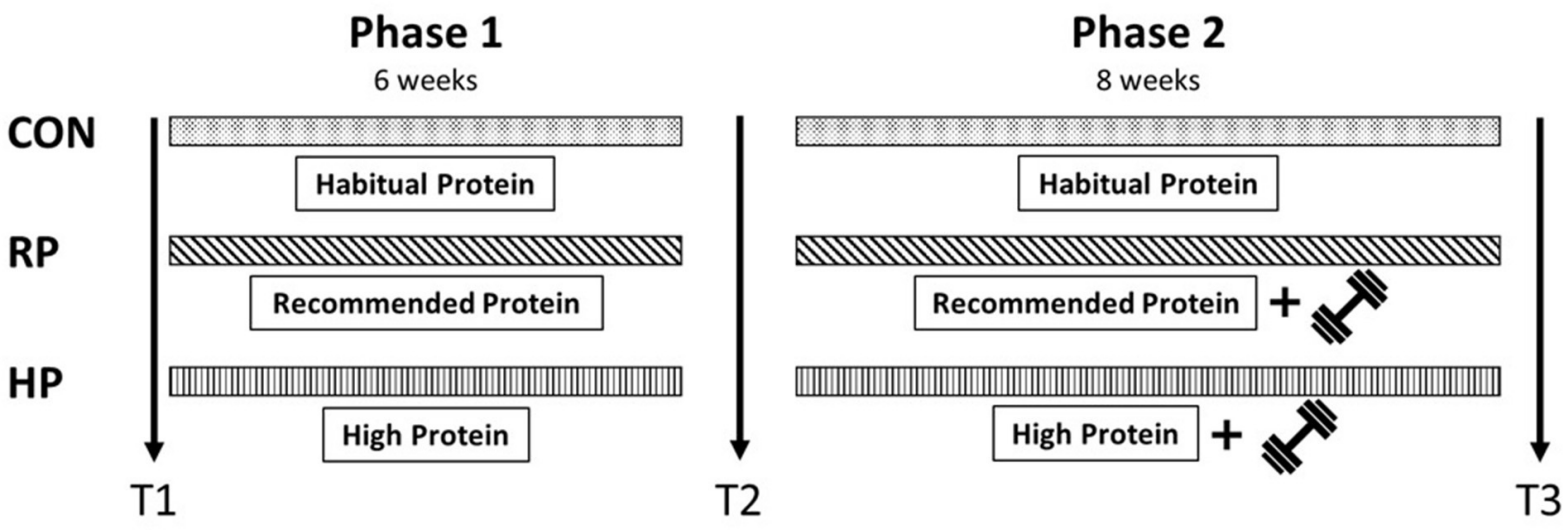
Study design.

Three groups, control group (CON, observation only), recommended protein intake plus resistance training group (RP) and high protein intake plus resistance training group (HP), were built, based on a randomized, controlled, observer-blind study design. Study participants were initially screened for in- and exclusion criteria by our medical team. Baseline testings (T1) were followed by a 6-week intervention focussing on the nutritional intervention only (phase 1) by either implementing a diet high in dietary protein, with 2.0 g per kg bodyweight per day (g/kg BW/d), a diet with a dietary protein intake according to the recommendations in the German speaking countries (1.0 g/kg BW/d), or controlling the habitual diet of CON group. Interim testings (T2) followed 8 weeks of nutritional intervention combined with a progressive resistance exercise training programme (phase 2) in RP and HP. The final examinations were performed in study week 17 (T3); see study design (Figure 1). All assessments were conducted at the Center for Sport Science and University Sports, University of Vienna, Austria between July and December 2018.

### Participants

Study participation was possible, if participants were between 65 and 85 years of age and did not conduct regular resistance training during the last 6 months before study entry. Study subjects were recruited *via* media announcements (local newspapers, online articles) and information events at local senior citizens meeting centers between June and September 2018. All interested candidates were screened regarding cognitive impairment (Mini Mental State Examination Score < 23), acute and chronic diseases that would contraindicate resistance training, serious cardiovascular diseases, diabetic retinopathy, manifest osteoporosis, anticoagulant or cortisone medication, frailty index (≥3) or the need for walking aids, all of which were exclusion criteria. Pre-existing conditions, such as hyperlipidaemia, diabetes mellitus type 2, osteoporosis, cardiovascular diseases and history of cancer, were registered during the initial medical examination, however, were acceptable for study participation, as long as they did not directly interfere with the physical performance tests and/or the exercise program.

All participants had to provide a written informed consent form in order to be part of the study. The study was performed in accordance with the Declaration of Helsinki, approved by the Ethics Committee of the University of Vienna (Reference Number: 00322) and registered at https://clinicaltrials.gov (NCT04023513).

### Interventions

#### Nutritional intervention

A food-based, personalized intervention was provided for the participants of the RP and HP groups. Based on regular, individual dietary assessments, a protein intake of 1.0 g/kg BW/d [D-A-CH reference values (19)] for the RP group, or 2.0 g/kg BW/d for the HP group was aimed for. Participants of the CON group did not achieve any nutritional intervention, but were encouraged and controlled to maintain their habitual diet over the whole study period. The participants of the RP and HP groups were regularly provided with commercially available whole food products, either rich in protein (e.g., protein-rich milk products, bars, puddings, protein-rich bread, bacon crisps, protein rich soups, pea protein sticks as well as recipes for self-prepared foods) or similar foods with regular protein content (e.g., milk products, bars, bread, soups, self-made vegetable muffins). The individual protein intake was calculated based on the participant's BW, daily recorded by the respective participant and monitored throughout the whole study period.

Starting with the exercise intervention, the HP group additionally received 40 g of veganeo hazelnut-chocolate drink [AnovonA^®^, Laufen, Germany: 146 kcal, 1.5 g fat, 1.4 g carbohydrates 32 g protein (leucine 3.1 g, isoleucine 1.6 g, valine 2.0 g)] or 40 g veganeo vanilla [AnovonA^®^, Laufen, Germany: 146 kcal, 1.1 g fat, 1.2 g carbohydrates, 32 g protein (leucine 3.2 g, isoleucine 1.7 g, valine 2.1 g)] dissolved in 300 ml water immediately post-exercise, while still continuing their dietary regimen. To compensate for this additional caloric supply, the RP group received an equivalent isocaloric, carbohydrate-containing drink [40 g bulkwoders.com, pure series (Colchester, UK): 152 kcal per serving; 0 g fat, 38 g carbohydrates (1:1, cyclical dextrin: dextrose & maltodextrin), 0 g protein with 300 ml water] after each training sessions. A physical activity level (PAL) between 1.4 and 1.6 (females: 1.700–1.900 kcal/d; males: 2.100–2.500 kcal/d) was estimated to calculate total energy expenditure, the target range for total energy intake for HP and RP groups was based on the D-A-CH reference values for this age group.

#### Resistance training

The exercise training program was performed between T2 and T3 for 8 weeks, twice a week on non-consecutive days following the guidelines of the American College of Sport Medicine (ACSM) (20). Both, the RP and HP groups, completed the same training sessions, consisting of 5 to 10 min of warm-up, followed by 45–60 min of resistance exercise training for the major muscle groups. The sessions finished with 5–10 min of cool-down. Commercial gyms, all equipped with the same training devices (TECHNOGYM Selection 700 & 900, Italy), were selected to have similar training conditions for all participants. The resistance training consisted of machine-guided exercises, free weights, as well bodyweight exercises (leg press, leg curl, latissimus pulldown, rowing, chest press, goblet box squat, dumbbell shoulder press, front plank with alternating single leg raise). Tempo was predefined to spend 1–2 s on the concentric and 3–4 s on the eccentric phase. Training intensity was subjectively rated by the study participants by using the OMNI Rate of Perceived Exertion scale (RPE 0–10) (20, 21). During the first 2 weeks, the participants performed one to two sets of 15–20 repetitions at submaximal load (RPE 3–4) for familiarization. From week three, the subjects performed two sets, increased the weight and decreased the number of repetitions to 10–15 with an RPE of 4–6. From week 6, the intensity was further increased to an RPE of 6–7, whereby the number of repetitions was decreased to 8–12 repetitions and the number of sets was increased to three. Sport scientists guided and supervised the training sessions, monitored the correct and safe execution of the exercises and adapted the intensity if necessary.

### Outcome parameters

Dietary, physical and medical assessment were performed during study weeks 1, 8 and 17. Trained research staff was blinded regarding group allocation of the participants and followed standardized procedures.

#### Dietary intake assessment

Data regarding dietary intake were collected by performing 24-h recalls every 7–10 days over the whole study period. On average, 9 ± 1 interviews per participant were conducted. Interview data were analyzed within 2 days to evaluate and if necessary adapt the participant's diet. At baseline, two 24-h dietary recalls were performed within 10 days. Seven out of nine interviews referred to weekdays while two were based on weekend days. Minimum the first four interviews were performed face-to-face. For the following assessments, participants could choose between face-to-face or a telephone interview. The interviews were guided by the software Globodiet^®^, formerly EPICSoft. GloboDiet was developed by the International Agency for Research on Cancer and further adapted for Austria at the Department of Nutritional Sciences in Vienna (22). To support the estimation of portion size, every participant received a photobook. The consumed foods were linked to the German food composition database (Bundeslebensmittelschlüssel) version 3.02 (23). Total energy intake (kcal), carbohydrates (g/kg BW/d), fat (g/kg BW/d) and protein (g/kg BW/d) were estimated. Additionally, the consumption of provided foods was reported in a food diary, which was reviewed and collected during the 24-h recalls.

#### Anthropometry and body composition

Anthropometric and body composition measurements were performed in the morning after an overnight fast. A stadiometer and digital scale (seca 217 + 877, Seca GmbH & Co KG, Hamburg, Germany) was used to measure body height (to 0.01 m) and mass (to 0.1 kg). Participants were lightly clothed and barefoot. Body mass index (BMI) was calculated—ratio of body weight (in kg) and height (in m) squared.

Body composition was measured with body impedance analysis (BIA, Nutriguard-MS + NutriPlus-Software, Version 5.1, Data-Input, GmbH, Germany). Data output included resistance (R, Ω), reactance (Xc, Ω), fat mass (kg and percentage) and phase angle (°). Skeletal muscle mass (SM) was calculated: SM {kg) = [(Ht^2^ /R^*^0.401) + (sex ^*^ 3.825) + (age ^*^ −0.071)] + 5.102}; where Ht is height in cm, R is resistance in Ω; sex = 1 for men and sex = 0 for women, and age is indicated in years (24).

### Plasma proteomics

Following an overnight fast, blood samples were collected into EDTA-coated vacutainers. Samples were placed in the fridge prior to separation of plasma for analysis, and then separated into aliquots and stored in Eppendorf tubes at −80°C until required for analysis.

#### Sample preparation

Plasma samples were diluted 1:10 in lysis buffer (8 M urea, 50 mM TEAB, 5% SDS), heated at 90°C for 5 min prior the protein concentration was determined using a BCA assay. For enzymatic protein digestion, 20 μg of protein was used and the ProtiFi S-trap technology (25) applied. In short, solubilized protein was reduced and carbamidomethylated by adding 64 mM dithiothreitol (DTT) and 48 mM iodoacetamide (IAA), respectively. Before loading the samples onto S-trap mini cartridges, trapping buffer (90 % v/v methanol, 0.1 M triethylammonium bicarbonate) was added. Thereafter, samples were thoroughly washed and subsequently digested using Trypsin/Lys-C Mix at 37°C for 2 hours. Finally, peptides were eluted, dried and stored at −20°C until LC-MS analyses.

#### LC-MS/MS analyses

Reconstitution of dried peptide samples was achieved by adding 5 μl of 30 % formic acid (FA) containing 4 synthetic standard peptides and subsequent dilution with 40 μl of loading solvent (97.9% H_2_O, 2% ACN, 0.05% trifluoroacetic acid). Thereof, 500 nL were injected into the Dionex Ultimate 3000 nano high performance liquid chromatography (HPLC)-system (Thermo Fisher Scientific). A pre-column (2 cm × 75 μm C18 Pepmap100; Thermo Fisher Scientific) run at a flow rate of 10 μl/min using mobile phase A (99.9 % H_2_O, 0.1% FA) was used to pre-concentrate peptides prior to chromatographic separation. Peptides were then separated on an analytical column [25 cm × 75 μm 25 cm Aurora Series emitter column (Ionopticks)] by applying a flow rate of 300 nL/min and using a gradient of 7–40 % mobile phase B (79.9% ACN, 20% H_2_O, 0.1% FA) over 43 min, resulting in a total LC run time of 85 min including washing and equilibration steps. Mass spectrometric analyses were performed using the timsTOF Pro mass spectrometer (Bruker) equipped with a captive spray ion source run at 1,650 V. Additionally, the timsTOF Pro mass spectrometer was operated in the Parallel Accumulation-Serial Fragmentation (PASEF) mode and a moderate MS data reduction was applied. Further parameters included a scan range (m/z) from 100 to 1,700 to record MS and MS/MS spectra and a 1/k0 scan range from 0.60 to 1.60 V.s/cm^2^ resulting in a ramp time of 100 ms to achieve trapped ion mobility separation. All experiments were performed with 10 PASEF MS/MS scans per cycle leading to a total cycle time of 1.16 s. Furthermore, the collision energy was ramped as a function of increasing ion mobility from 20 to 59 eV and the quadrupole isolation width was set to 2 Th for m/z <700 and 3 Th for m/z > 700.

### Statistics

#### Sample size

The sample size calculation for this study was performed for the primary outcome parameter 30-s-chairstand test, as previously shown by Unterberger et al. (26). Based on these calculations a total number of about 130 subjects was aimed to be recruited into the study.

#### Randomization and stratification

After having been controlled for in- and exclusion criteria, participants were randomly assigned to one of the three intervention groups (1:1:1) by using an academic randomization tool and random permuted blocks (6, 3; https://randomizer.at/, Institute of Medical Informatics, Statistics and Documentation, Medical University of Graz, Austria). Subjects were stratified by age groups (65–69.9; 70–74.9; 75–79.9; 80 to <85 years) and sex (female; male) to achieve similar baseline conditions.

#### Statistical analyses

Data acquisition and data analyses were performed using commercial software with data files coded and anonymized. All statistical analyses were done using SPSS (IBM Corp, New York, NY, USA, version 26). One-way ANOVA and Chi-square tests were used to compare differences between groups at baseline. Main time and group effects as well as time^*^group interactions were determined using a two-way-mixed ANOVA with Bonferroni-corrected *post-hoc* tests. Data are expressed as mean [95% confidence intervals, or standard deviation]. Significance was set to α = 0.05.

#### LC-MS/MS data analysis

Protein identification as well as label-free quantification (LFQ) was achieved using the publicly available software package MaxQuant 1.6.17.0 running the Andromeda search engine (27). Therefore, raw data were searched against the SwissProt database (version 141219 with 20380 entries). Further, search parameter included an allowed peptide tolerance of 20 ppm, a maximum of 2 missed cleavages, carbamidomethylation on cysteins as fixed modification as well as methionine oxidation and N-terminal protein acetylation as variable modification. A minimum of one unique peptide identifications per protein was used as search criterium. In addition, the “match between runs” option was applied, using a 0.7 min match time window and a match ion mobility window of 0.05 as well as a 20 min alignment time window and an alignment ion mobility of 1. An FDR ≤ 0.01 was set for all peptide and protein identification.

Subsequent data evaluation and statistical analysis was accomplished using the Perseus software (version 1.6.14.0) (27). Identified proteins were first filtered for reversed sequences as well as common contaminants and annotated according to sampling time point and intervention group. Prior to statistical analysis, proteins were additionally filtered for their number of independent identifications (70% in at least one group). For paired *t*-tests, only participants providing plasma samples for each time point were considered. Both, two-sided paired *t*-tests as well as unpaired *t*-test statistics for volcano plots were performed applying an FDR of 0.05 and a S0 of 0.1, whereby S0 controls the relative importance of *t*-test *p*-value and difference between the means. Histograms and heat maps were generated using GraphPad Prism Version 6.07 (2015) and RStudio Version 1.4.1717.

#### Missing data

At baseline dietary data from nine participants, who left the study already before their first 24-h recall are missing. One participant could not be interviewed within the first 2 weeks. Consequently, nutritional data from 124 subjects were analyzed at baseline. Furthermore, baseline body composition data are missing from five participants due to technical issues. Plasma samples for proteomics analyses were available for all participants.

## Results

### Participants' flow

**Figure 2:**
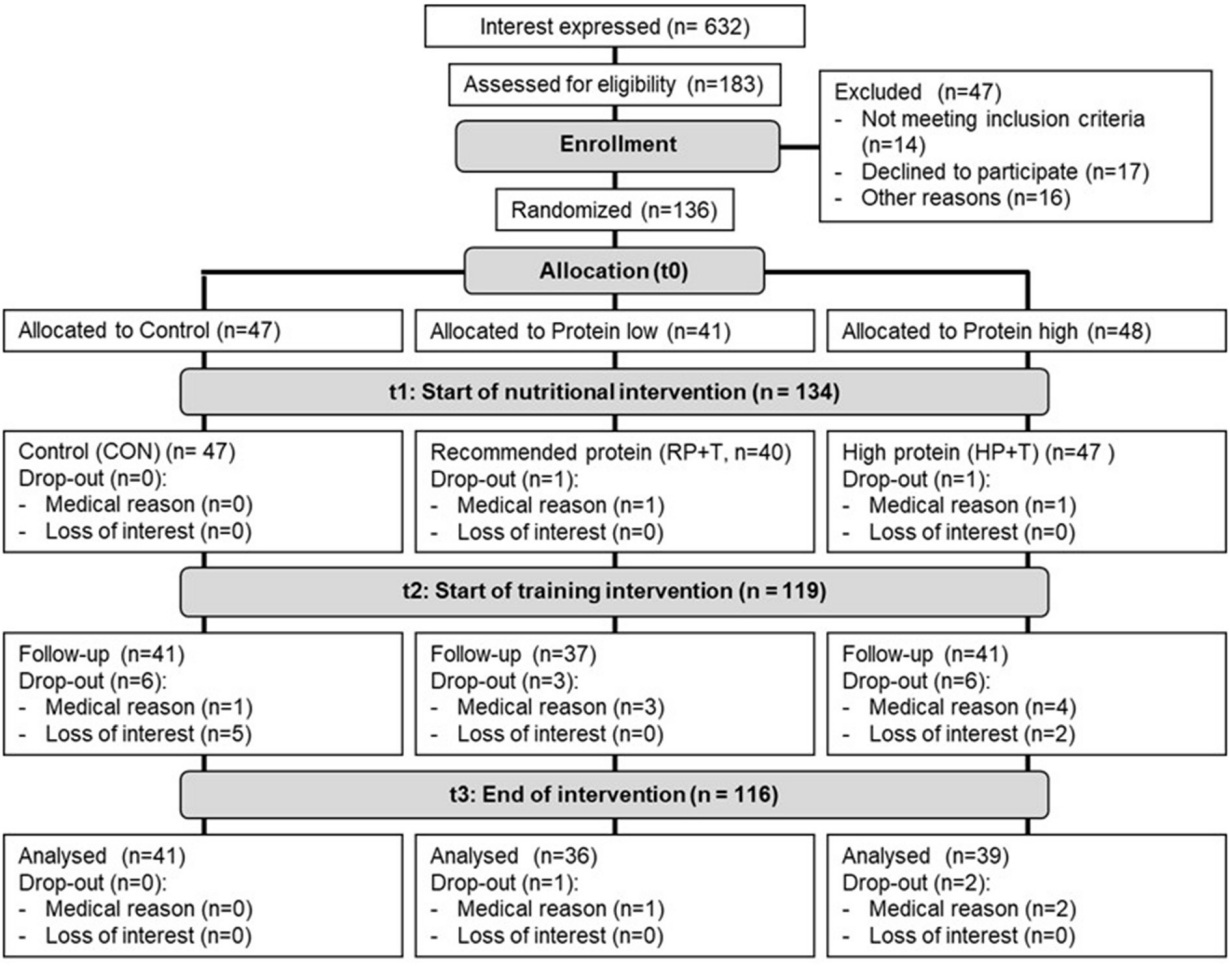
Participants' flow.

A total of 632 people declared to be interested in the study and 183 underwent the medical pre-examination. Finally, 136 persons met the eligibility criteria and were randomly allocated to CON (*n* = 47), RP (*n* = 48) and HP (*n* = 41). Two participants withdrew due to medical reasons after randomization and before t1. Finally, 134 individuals were included in the baseline assessments and represented the final study population. In the nutritional intervention phase, from t1 to t2, fifteen people dropped out of the study (CON: *n* = 41, 87.2%; RP: *n* = 37, 92.5%; HP: *n* = 41, 87.2%). A total of 116 (86.5%) participants (CON: *n* = 41, 87.2%; RP: n = 36, 90.0%; HP: *n* = 39, 83.0%) finished the study. None of the dropouts left the study due to injury or adverse reactions to the interventions and there was no difference between the groups (*p* = 0.602). Details of the participant flow are shown in Figure 2.

### Baseline characteristics

Baseline characteristics for anthropometric measurements, macronutrient intake, body composition, blood lipid profile and CRP are shown in Table 1.

**Table 1:** Baseline characteristics.

	**Total**	**Female**	**Male**	***p*** **-value**
Sex [f/m] (% females)]	134	72 (53.7)	62 (46.3)	0.435
Age [years]	72.9 ± 4.8	73.0 ± 4.7	72.7 ± 5.0	0.778
Body mass [kg]	74.3 ± 13.6	67.2 ± 11.9	82.3 ± 10.9	**<0.001**
Height [m]	1.68 ± 0.1	1.62 ± 0.6	1.76 ± 0.7	**<0.001**
BMI [kg/m^2^]	26.2 ± 3.9	25.7 ± 4.2	26.6 ± 3.6	0.175
Energy intake [kcal/d]^*^	1,845 ± 714	1,623 ± 649	2,105 ± 703	**<0.001**
Protein intake [g/kg BW/d]^*^	0.85 ± 0.43	0.82 ± 0.52	0.89 ± 0.30	0.336
Protein intake [g/d]^*^	62.3 ± 29.6	53.6 ± 29.5	72.5 ± 26.5	**<0.001**
Fat intake [g/d]^*^	78.2 ± 39.1	69.7 ± 34.9	88.3 ± 41.6	**0.007**
Carbohydrate intake [g/d]^*^	188.5 ± 83.7	166.6 ± 75.8	214.3 ± 85.7	**0.001**
Lean body mass [kg]^#^	56.3 ± 11.7	47.5 ± 5.6	67.1 ± 7.4	**<0.001**
Skeletal muscle mass [kg]^#^	25.5 ± 7.2	19.8 ± 3.0	32.6 ± 3.7	**<0.001**
Body fat [kg]^#^	18.0 ± 7.1	19.9 ± 7.5	15.7 ± 5.7	**0.001**
Body fat [%]^#^	24.2 ± 7.6	28.6 ± 6.2	18.6 ± 5.1	**<0.001**
Total-C [mmol/L]	11.37 ± 2.30	12.07 ± 2.05	10.55 ± 2.33	**<0.001**
LDL-C [mmol/L]	6.52 ± 2.07	6.94 ± 2.02	6.04 ± 2.03	**0.011**
HDL-C [mmol/L]	3.57 ± 0.92	3.88 ± 0.91	3.20 ± 0.79	**<0.001**
TG [mmol/L]	6.36 ± 2.75	6.27 ± 2.94	6.47 ± 2.51	0.674
CRP [mg/l]	2.18 ± 2.40	2.26 ± 2.11	2.09 ± 2.71	0.675

*Values are shown as mean ± stdv. p-Values refer to differences between female and male participants (one-way ANOVA, Chi Square Test). BMI, body mass index; C, cholesterol; TG, triglycerides; CRP, c-reactive protein*.

* *n = 124;*

# *n = 129. Significant differences were highlighted bold*.

### Intervention effects

The dietary intervention in the RP and the HP groups resulted in an increased protein intake in both groups. The RP group increased the protein intake from 0.89 ± 0.28 g/kg BW/d up to 1.09 ± 0.33 g/kg BW/d at T2 and 1.06 ± 0.26 g/kg BW/d at T3. The participants of the HP group started the intervention at a protein intake of 0.80 ± 0.31 g/kg BW/d and increased the intake up to 1.54 ± 0.35 g/kg BW/d and 1.63 ± 0.36 g/kg BW/d, at T2 and T3, respectively. The CON group remained their protein intake stable over the whole study period between 0.83 and 0.91 ± 0.41 g/kg BW/d. Regardless of the intervention group, neither carbohydrate nor dietary fat intake changed during the intervention. Although, a significant increase in absolute energy intake in the HP group was observed, this effect could not be seen after correcting for bodyweight.

**Figure 3:**
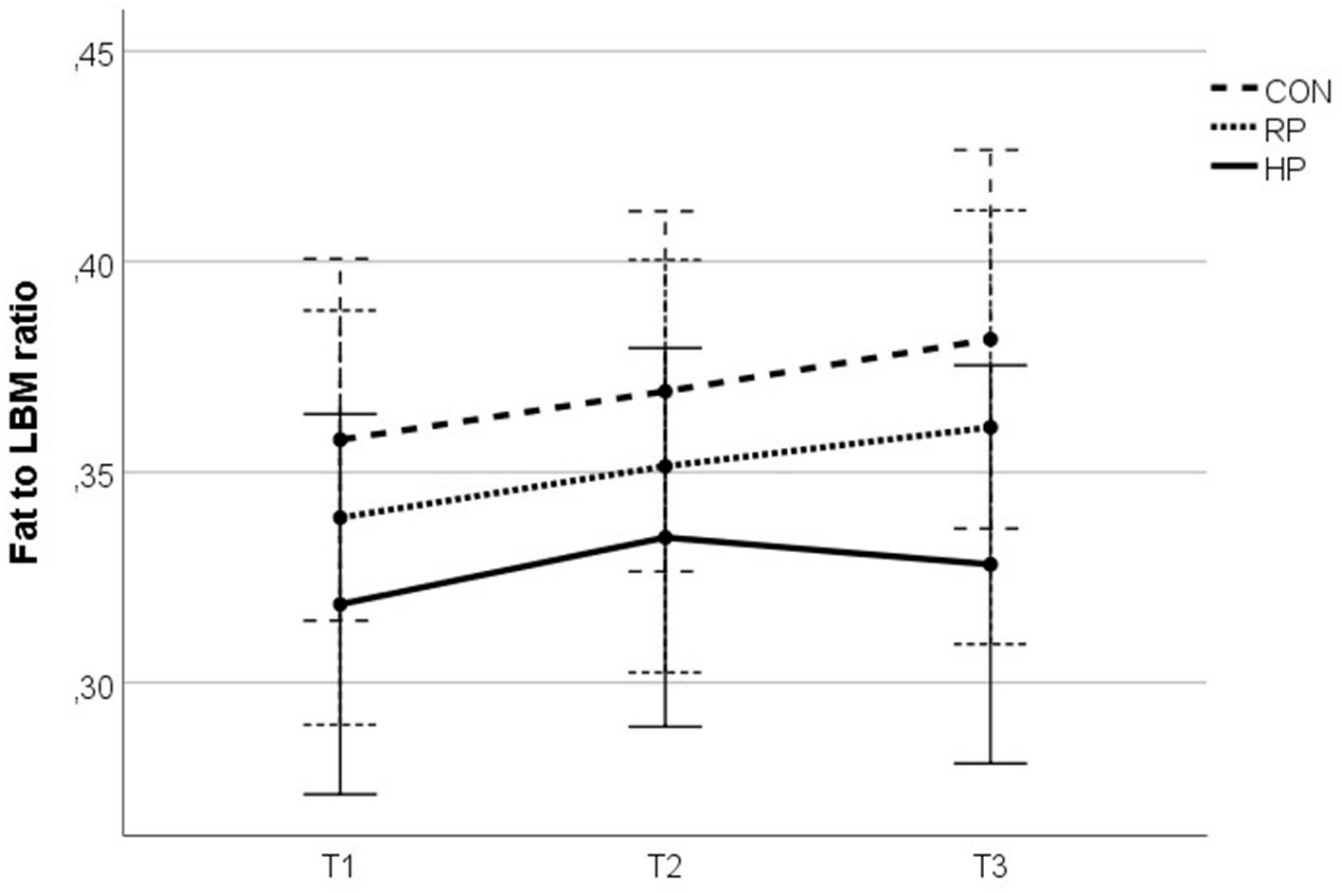
Fat to lean mass ratio over the study duration.

**Figure 4:**
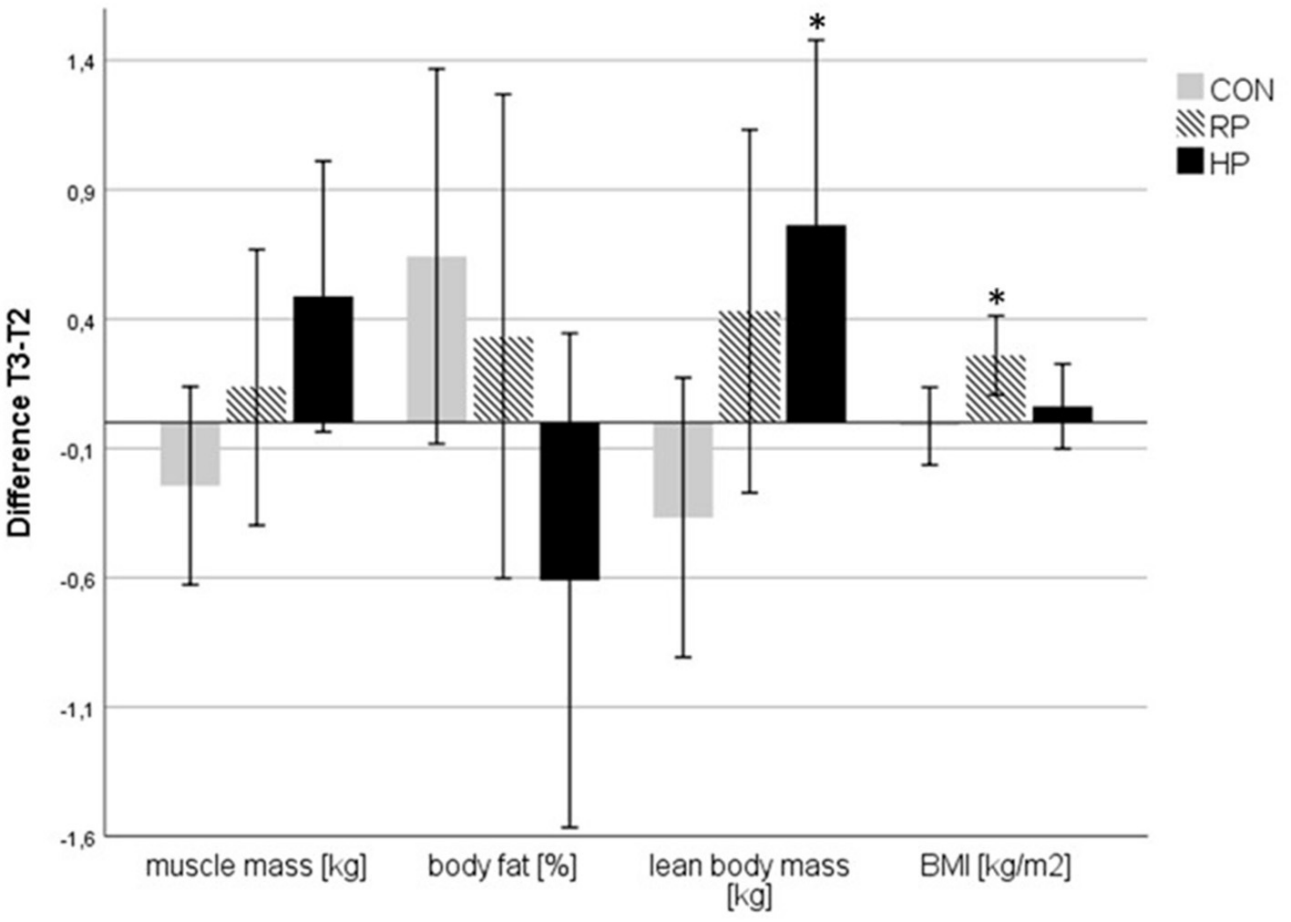
Changes in body composition parameters between T2 and T3. *p < 0.05 and indicates a significant difference to CON.

After 17 study weeks, we observed significantly increased body fat (+1.04 ± 2.20 kg) and at the same time a reduction in muscle mass (−0.24 ± 1.24 kg) in the CON group. While the RP group demonstrated a consistent increase in body fat (+1.2 ± 2.69 kg) over the whole study period, participants of the HP group showed a reversal of this trend in phase 2 of the study (combined exercise and dietary intervention, see Figures 3, 4). BMI increased significantly in the RP group. For details please see Supplementary Table 1 and Unterberger et al. (26).

The fat to lean body mass (LBM) ratio (Figure 3) summarizes the above described effects of the intervention on the participants' body composition and illustrates the reversed dynamic in the HP group, which was observed in phase 2 of the study.

To further investigate this observation, we calculated the differences (deltas) between T2 and T3 for muscle mass, body fat, LBM and BMI for the combined exercise and dietary intervention phase (Figure 4). For LBM we detected a significant difference between HP and CON group, whereas for BMI a significant difference between RP and CON was seen.

### Effects of dietary intervention and resistance training on the plasma proteome

**Figure 5:**
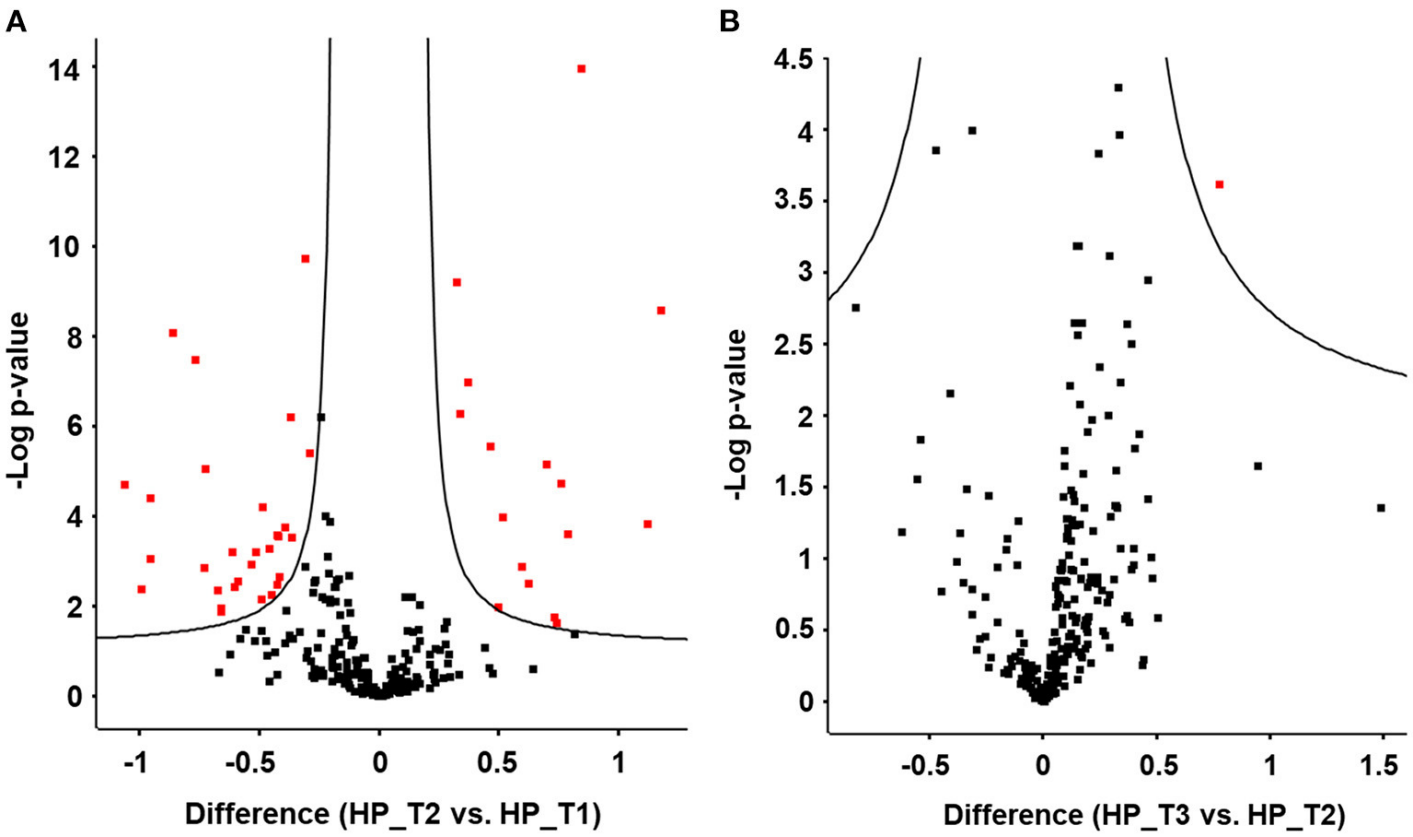
Plasma proteome analysis reveals significant changes in protein abundance upon high protein diet (HP). Volcano plots display significant protein changes (red) upon **(A)** high protein diet between T1 and T2 and upon **(B)** resistance training between T2 and T3 in the HP group. Volcano plots visualize results from unpaired *t*-test statistics. Differences are plotted as logarithmic values to the basis of 2.

LC-MS/MS-based plasma proteome analysis revealed a total number of 255 identified proteins after applying stringent filter criteria (see Material and methods). Comparative proteomics of the different intervention groups and phases was performed in order to identify potential adaptive responses. Paired *t*-test statistics between T1 and T2 in the high protein group revealed 68 significantly regulated plasma proteins (Figure 5A). Additional resistance training between T2 and T3 in the high protein group showed hardly any changes in the plasma proteome (Figure 5B). Comparison of protein abundance between T1 and T2 of the recommended protein as well as the control group lead to the identification of 90 and 80 significantly regulated proteins, respectively. Resistance training (T2 vs. T3) in these groups again had less effect on the plasma protein levels (Supplementary Table 2 and Supplementary Figure 1).

**Figure 6:**
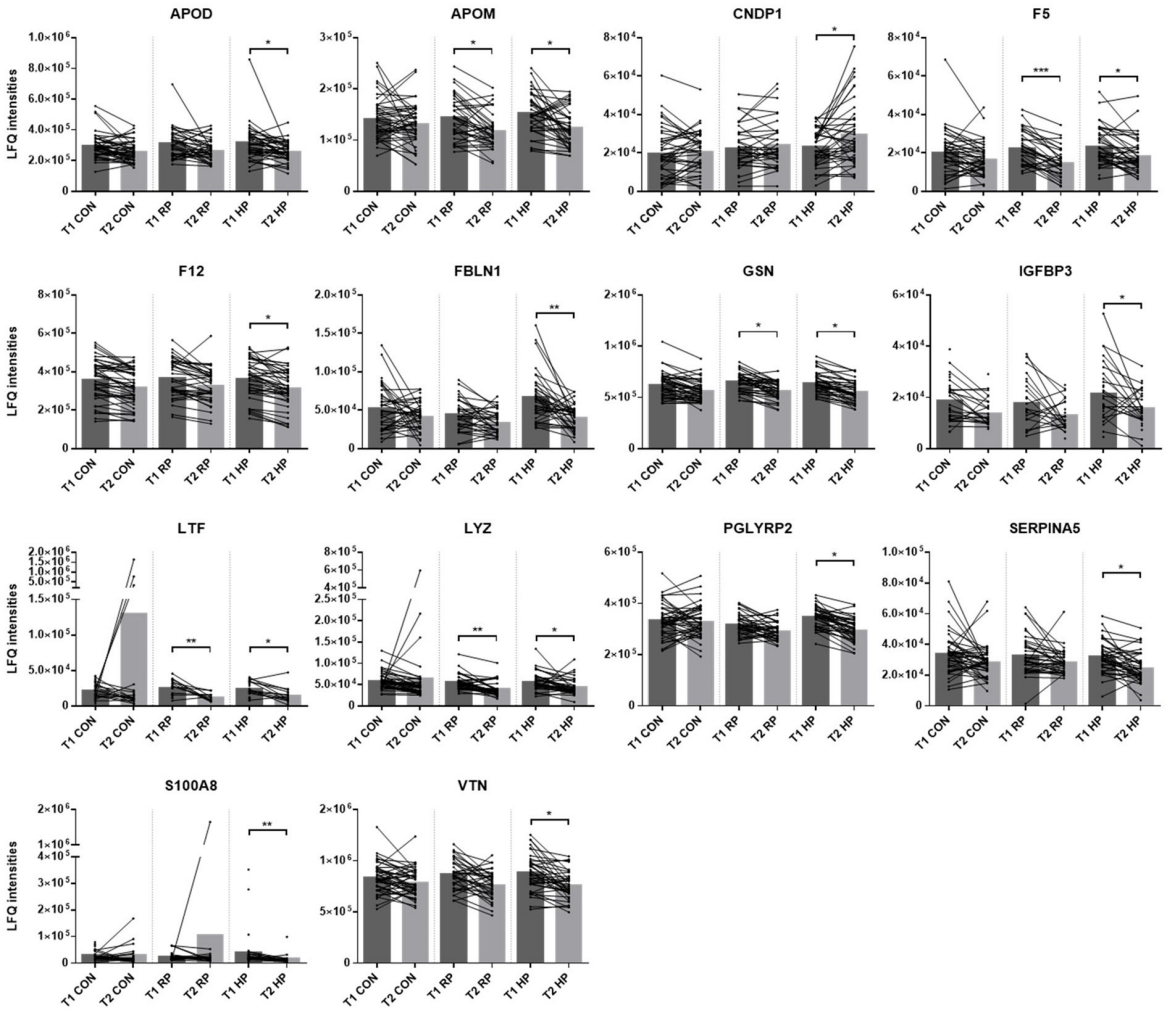
Proteins specifically regulated upon high protein dietary intervention. Histograms display label-free quantification values (LFQ intensities) of proteins, significantly regulated upon dietary intervention in the high protein group. Significant changes between T1 and T2 within the control group (CON), the recommended protein group (RP) as well as the high protein group (HP) are marked with asterisks. *q-value ≤ 0.05, **q-value ≤ 0.005 and ***q-value ≤ 0.001. Apolipoprotein D (APOD), apolipoprotein M (APOM), Beta-Ala-His dipeptidase (CNDP1), coagulation factor V (F5), coagulation factor XII (F12), fibulin 1 (FBLN1), gelsolin (GSN), insulin like growth factor binding protein 3 (IGFBP3), lactotransferrin (LTF), lysozyme C (LYZ), N-acetylmuramoyl-L-alanine amidase (PGLYRP2), plasma serine protease inhibitor 5 (SERPINA5), protein S100-A8 (S100A8), vitronectin (VTN).

In order to determine significant protein regulations specifically associated with high protein dietary intervention, the originally 68 significant proteins were filtered to exclude those proteins found to be also significant between T1 and T2 in the control group as well as four immunoglobulins. This resulted in the identification of 14 proteins (apolipoprotein D, apolipoprotein M, Beta-Ala-His dipeptidase, coagulation factor V, coagulation factor XII, fibulin 1, gelsolin, insulin like growth factor binding protein 3, lactotransferrin, lysozyme C, N-acetylmuramoyl-L-alanine amidase, plasma serine protease inhibitor 5, protein S100-A8, vitronectin) specifically regulated upon dietary intervention in the HP group (Figure 6).

**Figure 7:**
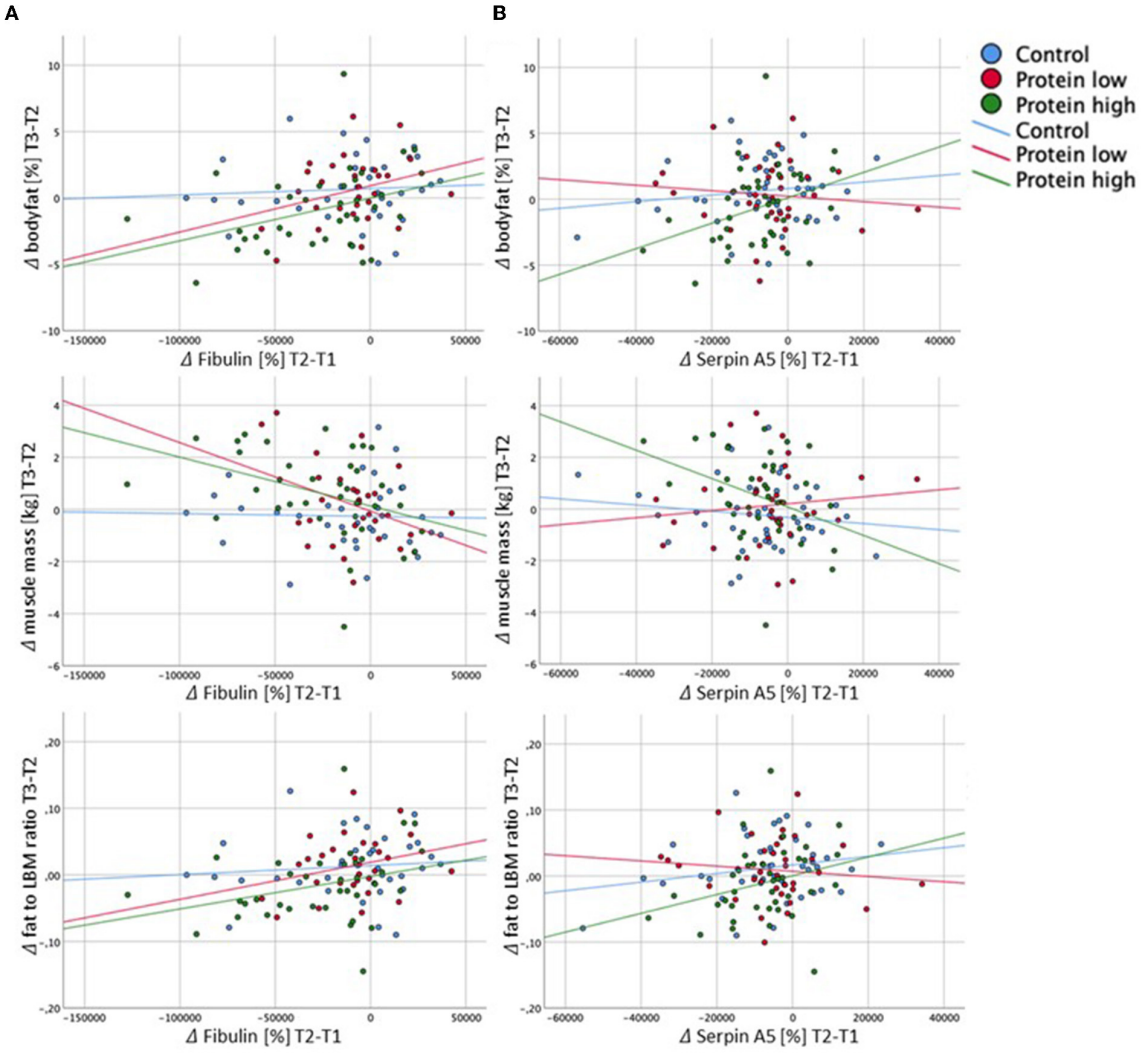
Correlations of deltas (Δ) from fibulin **(A)** and serpin A5 **(B)** with body composition parameters. **(A)** Significant and borderline-significant correlations of delta fibulin (T2-T1): delta bodyfat (T3-T2): HP: *r* = 0.364, *p* = 0.029; delta muscle mass (T3-T2): RP: *r* = −0.367, *p* = 0.050; HP: *r* = −0.388, *p* = 0.019; delta fat to LBM ratio (T3-T2): HP: *r* = 3.05, *p* = 0.070. **(B)** Significant and borderline significant correlations of delta serpin A5 (T2-T1): delta bodyfat (T3-T2): HP: *r* = 0.348, *p* = 0.028; delta muscle mass (T3-T2): HP: *r* = −0.361, *p* = 0.022; delta fat to LBM ratio (T3-T2): HP: *r* = 0.280, *p* = 0.080.

The data above indicate that the dietary intervention in phase 1 mediated the main changes in the plasma proteome, specifically of the HP group (Figure 6), whereas no further changes were observed in phase 2 (Supplementary Table 2). The additional exercise intervention in phase 2 initiated favorable changes in body composition, again predominantly in the HP group. To further explore these findings, we correlated the deltas (difference between T2 and T3) of body fat, muscle mass and fat to LBM ratio with deltas (difference between T1 and T2) of the proteins above (Figure 6). We found significant correlations with fibulin and serpin A5, specifically in the HP group (Figure 7).

## Discussion

This secondary analysis of the NutriAging protein study (26) aimed at investigating the effect of three different diets—a high protein intake, a protein intake according to the recommendations, a habitual diet—alone and combined with resistance training on markers of body composition and the plasma proteome in elderly women and men (65–85 years).

Throughout the study period, participants of the CON group maintained their protein intake within the range of 0.8 to 0.9 g/kg BW/d, whereas the RP group increased their intake up to the recommended level (28) of about 1 g/kg BW/d. For the participants of the HP group, we managed to double the initial protein intake of 0.8 g/kg BW/d and achieved over 1.6 g/kg BW/d. By doubling the protein intake in our cohort of older adults, we respected recent reports, that showed a beneficial effect on body composition, as well as muscle function in the presence of significantly increased protein intakes in elderly subjects (11, 29). These observations go hand in hand with the data from Moore et al. (30) whose regression analysis indicate, that elderly individuals, compared to younger adults, need almost twice the amount of protein to similarly stimulate muscle protein synthesis.

In our study we observed a beneficial effect of a high protein diet on lean body mass and fat mass, yet only in phase 2 of the intervention, when the combined nutritional and strength training intervention was performed (Figures 3, 4). In phase 1, despite the different dietary protocols, all three study groups showed a similar pattern of reduced muscle mass and increased fat mass, also mirrored by an increased fat-to-LBM ratio (Figure 3). This observation may point to seasonal effects as the study started during summer time and lasted until winter (31). By following their habitual lifestyle, the body composition from the participants of the CON group further followed this unfavorable development in study phase 2, which underlined the negative effects of a more sedentary lifestyle, as CON group did not perform any exercise training. Although the subjects of the RP group performed the strength training protocol in phase 2, a similar development of the fat-to-LBM ratio, as compared to the CON group, was observed. Interestingly and contrary to what was seen in the CON group, the BMI of the RP group significantly increased in phase 2, caused by both, an increased fat mass, as well as higher LBM. Similar to the CON group, the BMI of the HP group remained unchanged, although the energy intake was similar as in the RP group. Both groups, RP and HP, slightly increased their overall energy intake (RP +153 kcal, HP +249 kcal); only the change in the HP group was significant to baseline.

Our observation of potential benefits for body composition of a diet high in protein has already been shown in elderly men (11), in weight-loss studies (32) as well as in trials emphasizing muscle gains (33). Specifically, the combined intervention of high protein diets and resistance exercise seems to be necessary to compensate for the aging-related anabolic resistance of human skeletal muscle tissue (34, 35).

In the context of aging, more and more data is emerging and supporting the concept of a close interplay between physical activity, diet, function and healthy aging (6). Together with other studies the present trial builds a strong case for the combined intervention of high(er) protein diets in combination with resistance training to successfully prevent the age-related loss of muscle mass and function, finally ending up in sarcopenia. In the clinical setting, muscle mass, specifically in older adults and/or frail patients, has already been reported as a predictive marker for disease and therapy outcome, as muscles constitute a large pool of amino acids, which are essential for optimal immune function and tissue repair (36, 37).

To gain deeper knowledge on the underlying mechanisms that were induced by our interventions we performed plasma proteomics analyses, which revealed interesting results, again predominantly in the HP group. We identified 14 proteins which were significantly affected during phase 1 of the study (nutritional intervention only), specifically in the HP group (Figure 6). Unexpectedly, no further changes of the plasma proteome were induced by the combined nutritional and exercise intervention.

High protein diet induced significant alterations of 14 plasma proteins, which may indicate a beneficial role of a high protein diet on the general health status of participants. The significantly regulated proteins point to three main biological processes: innate immune system, lipid modulation and transport as well as blood coagulation. Lactotransferrin (LTF), lysozyme C (LYZ) as well as Protein S100A8 belong to proteins secreted by immune cells such as neutrophils, macrophages and platelets and play an important role in the innate immune system (38–40). These proteins are significantly down-regulated upon HP diet indicating a lower basal level of inflammation-associated plasma proteins. Further, N-acetylmuramoyl-L-alanine amidase (PGLYRP2) as well as gelsolin (GSN) have both been already linked to inflammation and were found to be down-regulated in the HP group (41, 42).

The down-regulation of SERPINA5, a protein also displaying characteristics of acute phase proteins, as well as of apolipoprotein D (ApoD) and apolipoprotein M (ApoM) indicates a modulation of lipid-transporting proteins. Lower levels of ApoD and ApoM may point to a lower level of metabolic stress in the HP group since a high lipid metabolism has already been associated with higher metabolic stress (43). Especially the rise in ApoD levels has been linked to aging and neuropathologies (43–45). Moreover, ApoD was found to be enriched in high-density lipoproteins as well as blood plasma extracellular vesicles isolated from patients with coronary artery diseases (46). Thus, the significant down-regulation of proteins associated with lipid transport may not only indicate a generally lower level of metabolic stress but also a lower risk of developing cardiovascular disease in the HP group.

Furthermore, high protein diet may affect the coagulation system as demonstrated by the significant down-regulation of coagulation factor V and XII (F5 and F12). Both proteins are modulators of inflammatory responses and show higher levels in course of inflammatory processes (47, 48). Additionally, elevated blood levels of F12 are associated with the risk of vulnerable atherosclerotic plaques in the coronary arteries (49). Thus, the down-regulation of coagulation factors in the HP diet group may indicate a reduced thromboembolic risk. Concomitant with the down-regulation of the coagulation factors, significant lower levels of fibulin-1 (FBLN1) and vitronectin were observed upon high protein diet. Both proteins are associated with the extracellular matrix and have already been found to play an important role in pathophysiological processes leading to cardiovascular diseases and correlate with the vascular age (50–52). Moreover, changes in vitronectin levels were observed in response to calcification of the aortic valve (53) as well as in the course of breast cancer development (54). Interestingly, insulin-like growth factor-binding protein 3 (IGFBP3), a general disease marker, was also found to be significantly down-regulated upon HP diet. This finding was supported by published data as maternal high protein diet resulted in lower levels of serum IGFBP3 in weaning piglets (55). Additionally, IGFBP3 depletion leads to the suppression of tumor growth (56).

Beta-Ala-His dipeptidase (CNDP1), a carnosinase, is the only protein which was found significantly up-regulated upon high protein diet. This may indicate an adaptive response to changes in the diet. High protein diet might be associated with a higher demand for protease activity.

Conclusively, plasma proteomics strongly suggests a beneficial impact of the high protein diet on the innate immune system, lipid transport and coagulation system. Since all these processes are involved in chronic inflammation, amelioration thereof may result in reduced risk for age-related diseases like atherosclerosis, cancer and type 2 diabetes. It is well-established, that chronic low grad inflammation linked to aging impairs skeletal muscle protein synthesis, which in turn diminishes the adaptation to an exercise stimulus (57, 58). Interestingly, we observed significant correlations between changes in the two proteins fibulin1 and SERPINA5 in study phase 1 and changes in the three body composition parameters body-fat, muscle mass and fat-to-LBM ratio in study phase 2 (Figure 7), again predominantly in the HP group. We therefore suggest, that the high protein diet in phase 1, may have had systemic beneficial effects, which built a base of reduced inflammation and an improved metabolic state to further facilitate changes in body composition that were observed in study phase 2, after the combined nutritional and exercise intervention. Collectively, in this study a high protein diet significantly altered the protein expression pattern, but adding resistance training did not further change the plasma proteome. A future prospective study monitoring participants over a longer time period would be necessary to elucidate the long-term beneficial effect of a high protein diet in older adults.

This study has several strengths and limitations. To the best of our knowledge, the two-phased study design, with an initial nutritional phase and a following combined nutritional and exercise intervention has not been performed in this older adult cohort. Further, instead of only relying on protein supplements, we achieved a doubling of the baseline protein intake with a food-based approach, which is an important aspect regarding dietary adherence, specifically in this age-group. Although we individualized and tightly controlled the participants' diet, we cannot rule out inaccuracies in reporting. Yet, by performing 24-h recalls every 7–10 days we believe that we kept the reporting error at a minimum. As the intensity of the strength training program was controlled by subjective experience (RPE-scale), the resulting intensity was moderate, which we believe resulted in an extremely low dropout-rate during strength training intervention. Notably, the observed adaptations in physical functions and body composition could have been even more pronounced by controlling the exercise intensity *via* the individual repetition maximum. As mirrored by the drop-out rates and also the participants' feedback, the dietary intervention (drop-outs: *n* = 15) was the far bigger challenge, as compared to the exercise program (drop-outs: *n* = 3). In future studies, a more intensity focussed training program and targeting plasma proteins that originate from muscle tissue would open the window for further developing the emerging concept of “liquid biopsy” (59). Finally, the proteomics analyses were done in plasma but not in muscle tissue, therefore the results and their interpretation should be taken with caution.

## Conclusion

This randomized controlled trial in older women and men revealed numerous results which lead us to the following conclusions: First, a high protein diet in older adults seems to induce favorable changes in body composition, yet only when combined with resistance exercise. Second, the plasma proteome is significantly affected by a high protein diet, however, additional resistance training did not result in further adaptations of the proteome. Third, changes in the plasma proteome due to the high protein diet point to a beneficial impact on the innate immune system, lipid transport and coagulation system, all of which are involved in chronic disease development. Fourth, our observations indicate, that changes in the body composition, through exercise and a high protein diet, are possibly linked to changes in the plasma proteome, which in turn were predominantly induced by the high protein diet alone.

This study gained novel clinical data in the field of healthy, successful aging and further emphasizes the important combination of an optimized protein intake *via* foods and regular resistance exercise to improve body composition in older adults. Still, more work in the field of proteomics, linked with lifestyle intervention studies particularly in younger and older adults, is needed to identify, validate and link specific proteins and/or protein patterns to clinical characteristics.

## Data availability statement

The datasets presented in this study can be found in online repositories. The names of the repository/repositories and accession number(s) can be found below: http://www.proteomexchange.org/, TBA, ProteomeXchange, identifier PXD033493.

## Ethics statement

The studies involving human participants were reviewed and approved by Ethics Committee of the University of Vienna (Reference Number: 00322). The patients/participants provided their written informed consent to participate in this study.

## Author contributions

K-HW, BW, and BF designed research. BF, AB, SU, RA, PZ, and AD conducted research. AB and CG performed proteomics analysis. BF and AB analyzed data and wrote paper. K-HW had primary responsibility for final content. E-MS performed the medical examinations. All authors have read and approved the final manuscript.

## Funding

This work was supported by the University of Vienna, by funding the Research Platform Active Ageing and the EU-program Interreg SK-AT (NutriAging). This article was supported by the Open Access Publishing Fund of the University of Vienna.

## Conflict of interest

The authors declare that the research was conducted in the absence of any commercial or financial relationships that could be construed as a potential conflict of interest.

## Publisher's note

All claims expressed in this article are solely those of the authors and do not necessarily represent those of their affiliated organizations, or those of the publisher, the editors and the reviewers. Any product that may be evaluated in this article, or claim that may be made by its manufacturer, is not guaranteed or endorsed by the publisher.
