# Supplementary material for: The plasma proteome is favorably modified by a high protein diet but not by additional resistance training in older adults: A 17-week randomized controlled trial
Source: Front Nutr. 2022 Aug 5;9:925450. doi: 10.3389/fnut.2022.925450 (PMC9389340; doi:10.3389/fnut.2022.925450)
Supplement: Supplementary file 1 [file Table_1.DOCX]

Supplementary Material

Supplementary Table 1: Intervention effects on parameters for body composition.

| Parameter | Group | Mean (95 % confidence interval) | | | time  p-value | group  p-value | time x group  p-value |
| --- | --- | --- | --- | --- | --- | --- | --- |
|  |  | Baseline (T1) | 8 weeks (T2) | 17 weeks (T3) |  |  |  |
| Body weight [kg],  N = 116 | CON | 73.7 [69.5;78.0] | 74.2 [70.0;78.4] | 74.2 [70.0;78.5] | **<0.001** | 0.598 | **0.001** |
|  | RP | 75.8 [71.3;80.4] | 76.2 [71.8;80.7] | 77.2^***,°°°^ [72.6;81.7] |  |  |  |
|  | HP | 73.1 [68.7;77.5] | 73.2 [68.9;77.5] | 73.5 [69.1;77.8] |  |  |  |
| BMI [kg/m²], N = 116 | CON | 26.0 [24.8;27.2] | 26.2^*^ [25.0;27.4] | 26.2 [24.9;27.4] | **0.001** | 0.764 | **0**.**002** |
|  | RP | 26.4 [25.1;27.7] | 26.5 [25.2;27.8] | 26.7^***,°°°^ [25.4;28.0] |  |  |  |
|  | HP | 25.9 [24.7;27.2] | 25.8 [24.6;27.1] | 25.9 [24.6;27.1] |  |  |  |
| Body fat [%], N = 109 | CON | 24.8 [22.4;27.1] | 25.7^*^ [23.4;28.1] | 26.1^**^ [23.7;28.5] | **<0.001** | 0.764 | 0.592 |
|  | RP | 24.7 [22.1;27.3] | 25.4 [22.9;28.0] | 25.8 [23.1;28.4] |  |  |  |
|  | HP | 22.9 [20.6;25.3] | 24.3 [22.0;26.7] | 23.7 [21.3;26.2] |  |  |  |
| Body fat [kg], N = 109 | CON | 18.3 [16.1;20.6] | 19.2^*^ [17.0;21.4] | 19.4^**^ [17.1;21.7] | **<0.001** | 0.495 | 0.379 |
|  | RP | 18.7 [16.2;21.2] | 19.3 [16.8;21.8] | 19.9^*^ [17.4;22.4] |  |  |  |
|  | HP | 17.0 [14.7;19.2] | 18.0^*^ [15.7;20.2] | 17.5 [15.2;19.8] |  |  |  |
| SM [kg],  N = 109 | CON | 25.0 [22.7;27.3] | 24.6 [22.3;26.8] | 24.4^*^ [22.1;26.7] | **0.018** | 0.829 | 0.333 |
|  | RP | 25.6 [23.1;28.1] | 25.4 [22.9;27.9] | 25.6 [23.1;28.2] |  |  |  |
|  | HP | 25.9 [23.6;28.2] | 25.1 [22.8;27.4] | 25.6 [23.3;27.9] |  |  |  |
| Values are shown as mean [95 % CI]. p-Values refer to main effects of time and group as well as time*group interactions (two-way mixed ANOVA). Significant effects (p < 0.05) are shown in bold. CON (control group); RP (recommended protein group); HP (high protein group). In case of significant overall time effects, Bonferroni-corrected post hoc analyses were performed individually for groups, whereby asterisks indicate significant differences to t1 and circles to t2. ***^,°°°^ (p<0.001); **^,°°^ (p<0.01); *^,°^ (p<0.05). *Data are extracted from* *Unterberger et al. (26)*. | | | | | | | |
