# Supplementary material for: The plasma proteome is favorably modified by a high protein diet but not by additional resistance training in older adults: A 17-week randomized controlled trial
Source: Front Nutr. 2022 Aug 5;9:925450. doi: 10.3389/fnut.2022.925450 (PMC9389340; doi:10.3389/fnut.2022.925450)
Supplement: Supplementary file 3 [file Image_1.PDF]

Supplementary Material

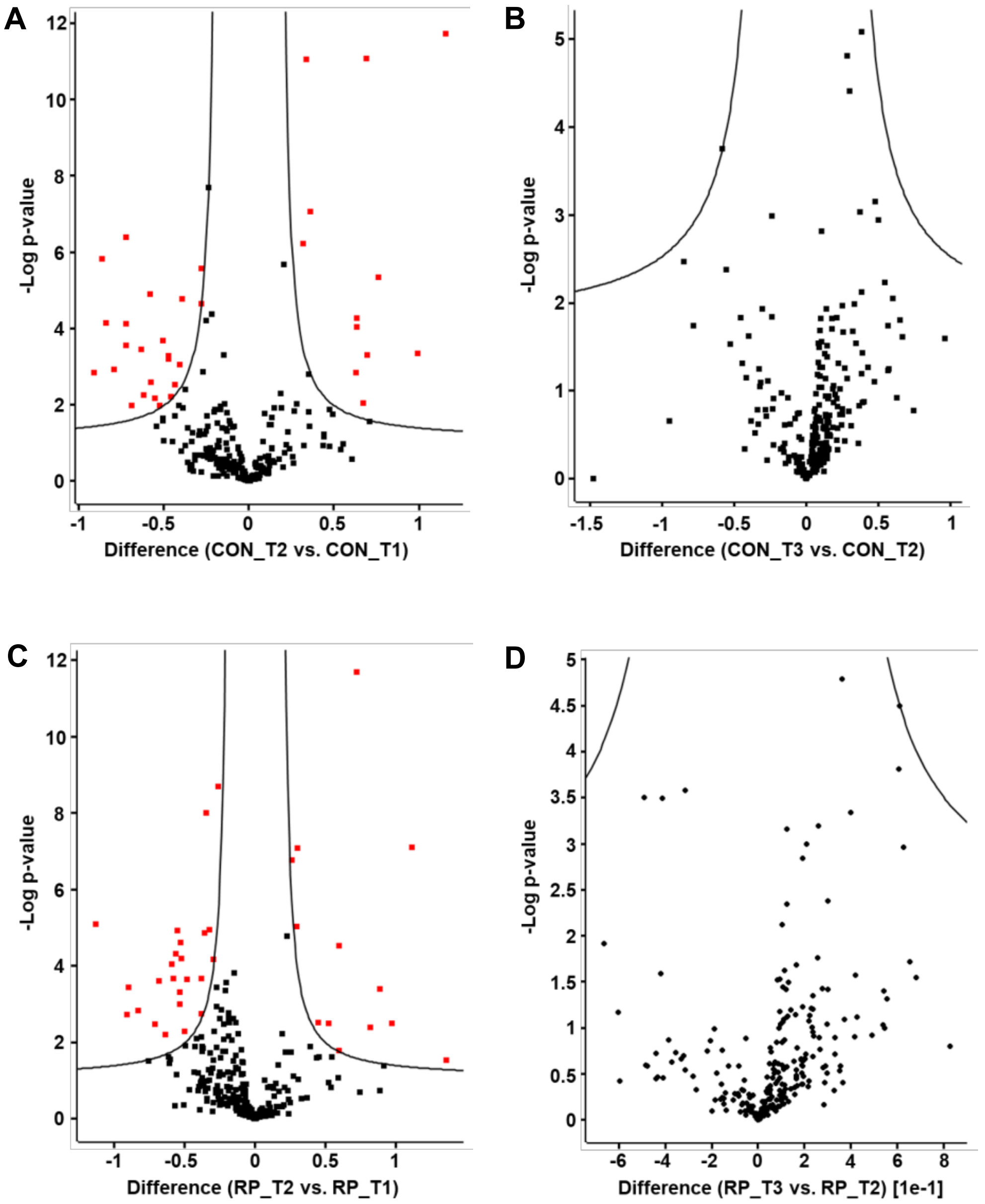

Supplementary Figure 1: Plasma proteome analysis reveals significant changes in protein abundance upon recommended protein intake (RP) as well as in the control group (CON). Volcano plots display significant protein changes (red) in the control (A) as well as in the recommended protein intake (C) study group between T1 and T2. Furthermore, protein abundance changes upon resistance training in the respective study group (B and D) are shown. Volcano plots visualize results from unpaired t-test statistics. Differences are plotted as logarithmic values to the basis of 2.
